# Practical Points

**Published:** 1892-05-14

**Authors:** 


### PRACTICAL POINTS.

Questions are invited for this column on any point in the administration of hospitals and asylums which may prove of difficulty or interest to our readers. We propose to give a few typical questions and answers, and shall continue them from time to time providing the idea is taken up by those for whose benefit it is chiefly intended. All communications should be marked "Practical Points," addressed to the Editor of The Hospital, 140, Strand, W.C.

*Abstract of Accounts.—"Ayr" writes: I shall be obliged if you can send me a copy of the most approved form of abstract of accounts.*

There is no better form of abstract of accounts than the analysis adopted by the Metropolitan Hospital Sunday Fund, particulars of which will be found in the next edition of "Burdett's Hospital Annual."

*Comparative Working of Certain Hospitals.—"Nottingham" asks: Will you be kind enough to furnish me with a statement showing the number of beds, average number of in-patients, number of in and out-patients, and average cost of each bed occupied in the following hospitals: Nottingham General Infirmary, Birmingham General Infirmary, Manchester Royal Infirmary, Royal Southern Hospital, Liverpool, Edinburgh Royal Infirmary, Staffordshire General Infirmary, North Stafford Infirmary, Newcastle-on-Tyne Royal Infirmary, Leeds General Infirmary, Bristol Royal Infirmary?*

The following table will give you the information you ask for:—

| Name of Hospital. | No. of beds. | Daily average No. occupied. | No. of in-patients | No. of out-patients | Average cost of each bed occupied. | | |
|---|---|---|---|---|---|---|---|
| Nottingham General ...... | 180 | 135 | 1,419 | 7,609 | £56 | 1 | 9 |
| Birmingham General...... | 280 | 213 | 3,607 | 45,950 | 57 | 14 | 11 |
| Manchester Royal ......... | 298 | 263 | 4,239 | 32,873 | 55 | 10 | 2 |
| Liverpool Southern ...... | 200 | 170 | 1,975 | 8,058 | 42 | 5 | 2 |
| Edinburgh Royal............ | 700 | 634 | 8,068 | 30,000 | 54 | 5 | 8 |
| Stafford Royal ................ | 124 | 87 | 1,460 | 16,972 | 57 | 10 | 5 |
| North Staffordshire ...... | 213 | 164 | 1,551 | 7,443 | 50 | 13 | 7 |
| Newcastle-on-Tyne Royal | 270 | 243 | 3,120 | 9,723 | 48 | 5 | 9 |
| Leeds General ............... | 320 | 290 | 4,814 | 29,834 | 52 | 2 | 1 |
| Bristol Royal Infirmary | 264 | 209 | 3,129 | 26,367 | 49 | 16 | 3 |

*Responsibility for the Custody of Would-be Suicides.—"R. M. O." writes: Do the police take charge of would-be suicides while in hospital, or is it left to the authorities of the institution to watch them, and hand them over to the police when recovered?*

The police are alone responsible for the custody of would-be suicides. The hospital authorities should do all in their power to aid the law, but they cannot resort to force or retain a patient against his will, unless the patient is in such a state as not to be responsible for his actions.

*Ready Printed Cash-books for Small Hospitals.—"Hon. Secretary" asks: Where can I obtain ready printed cash-books suitable for use in a small hospital?*

These cannot be procured ready printed, but suitable forms will be found in "Burdett's Hospital Annual."

*Analysis Journal for Hospital Use.—"I. of W." says: I shall be extremely thankful if you will send me a specimen of your Analysis Journal. I am anxious to introduce the system of accounts that Mr. Burdett advocates.*

See reply to "Ayr."

*Rules and Regulations for Matron and House Surgeon. "A. B." Torquay.—Will you advise me as to the best rules and regulations to define the duties of House Surgeon and Matron in a small hospital? We find very great friction exists. Should like to put a stop to it.*

Unless there is a good understanding between the two officers, and a determination on both sides to work harmoniously, rules and regulations defining their duties are not likely to counteract the evil that exists. The sooner the officers are parted, the better.

*Cubic Space for Nurses' Sleeping Apartments.—"Matron" asks: What should be the cubic space per bed in the nurses' room?*

Each nurse should be allowed 1,000 cubic feet whether sleeping in a separate bed-room or in a dormitory.

*Wood Floors.—"Provincial" asks: Is the application of paraffin to floors of wards approved of?*

The application of paraffin to ward floors is not generally approved of; it keeps the floors in good condition, but the strong smell caused by its use is much objected to by doctors, nurses, and patients.

